# A Fragment of the LG3 Peptide of Endorepellin Is Present in the Urine of Physically Active Mining Workers: A Potential Marker of Physical Activity

**DOI:** 10.1371/journal.pone.0033714

**Published:** 2012-03-23

**Authors:** Tony J. Parker, Dayle L. Sampson, Daniel Broszczak, Yee L. Chng, Shea L. Carter, David I. Leavesley, Anthony W. Parker, Zee Upton

**Affiliations:** 1 Tissue Repair and Regeneration Program, Institute of Health and Biomedical Innovation, Queensland University of Technology, Brisbane, Queensland, Australia; 2 Workforce Health Innovation Program, Institute of Health and Biomedical Innovation, Queensland University of Technology, Brisbane, Queensland, Australia; Ottawa Hospital Research Institute, Canada

## Abstract

Biomarker analysis has been implemented in sports research in an attempt to monitor the effects of exertion and fatigue in athletes. This study proposed that while such biomarkers may be useful for monitoring injury risk in workers, proteomic approaches might also be utilised to identify novel exertion or injury markers. We found that urinary urea and cortisol levels were significantly elevated in mining workers following a 12 hour overnight shift. These levels failed to return to baseline over 24 h in the more active maintenance crew compared to truck drivers (operators) suggesting a lack of recovery between shifts. Use of a SELDI-TOF MS approach to detect novel exertion or injury markers revealed a spectral feature which was associated with workers in both work categories who were engaged in higher levels of physical activity. This feature was identified as the LG3 peptide, a C-terminal fragment of the anti-angiogenic/anti-tumourigenic protein endorepellin. This finding suggests that urinary LG3 peptide may be a biomarker of physical activity. It is also possible that the activity mediated release of LG3/endorepellin into the circulation may represent a biological mechanism for the known inverse association between physical activity and cancer risk/survival.

## Introduction

The most common mechanism of musculoskeletal injury in the workplace is overexertion and this relationship has been mainly derived from injury incidence data and knowledge of the tasks involved [1], [2], [3]. As work is usually performed over 8 to12 hour periods (shifts) these types of analyses are limited in identifying the levels of activity and potential risk of overexertion and injury.

In sport, biomarkers have been used to evaluate factors which influence performance such as level of activity and fatigue in order to design training and workload strategies to enhance performance [4], [5], [6], [7]. Biomarker monitoring attempts to fine tune an athlete's training to induce an ‘over-reached’ state, rather than ‘over-trained’ state [8], [9]. An over-trained state is thought to be a stress response to excessive training loads and inadequate recovery [8]. It may also be described as staleness, overwork, burnout and chronic fatigue [10]. The results of biomarker analysis in elite athletes has therefore provided a model for examining exertion and fatigue in heavy industrial workers as similarities exist between the two situations regarding stimulus [11].

The stimuli for overtraining are multifactorial but generally involve excessive repetition of specific movement patterns which may be associated with both high and low magnitude forces [12]. When these loads exceed the repair capacity of musculoskeletal structures, acute or chronic injury and disorder may occur [13]. While muscle damage may be associated with the breakdown of muscle specific structural proteins and inflammation, connective tissue damage is associated with the effects of physical load on the extracellular matrix of musculoskeletal structures [14]. Furthermore, muscle damage after exercise has been confirmed objectively by myofibrilar disruption and inflammation, and indirectly as the perception of soreness and prolonged loss of strength and range of motion, and infiltration of leukocytes [15].

Many of the biomarkers used to monitor the influence of training and fatigue in athletes, focus on evaluation of stress responses, inflammation and altered metabolic states [4], [5], [6], [16]. Cortisol and urea are commonly selected as representative biomarkers of these conditions. Cortisol is often presented as a ‘stress’ hormone and also functions as an anti-inflammatory and catabolic enhancer in response to exercise [5]. Indeed, cortisol has been examined as an indicator of increased catabolic metabolism in construction workers exposed to 12 hr workdays and extended work weeks [17], and its circadian function has been shown to alter in response to shift work in nurses [18]. In addition, elevated blood urea levels are considered a measure of increased muscle protein breakdown and are generally increased in over-trained or fatigued athletes [19], [20]. While these biomarkers have not been extensively used to examine the effects of workload in the industrial setting, there is a need to evaluate these and identify more accurate indicators of exposure to physical exertion in the work environment. In regard to the later, protein profiling approaches such as SELDI-TOF MS have been applied to discover novel biomarkers of diseases, such as cancer [21], [22], cardiovascular [23], [24], and kidney disease [25].

Therefore, in this study we sought to investigate the potential influence of exposure to varying levels of physical activity in a cohort of mining workers by analysis of urinary cortisol and urea levels, and utilisation of a SELDI-TOF MS approach to examine urinary protein for potential biomarkers of physical activity.

## Methods

### Ethics Statement

This study was approved by Queensland University of Technology (QUT) Human Research Ethics Committee (HREC) (HREC # 3787H and amendment 060000 0188). Participants completed an informed consent form and a questionnaire concerning general health information, exercise habits, perceptions of work activity levels and recent history of musculoskeletal injury and disorder. Only subjects who reported that they did not have any diagnosed pre-existing medical condition, and were deemed fit for work under Australian workplace regulatory standards were included in the study. Each participant also provided 3 urine samples.

### Preparation and engagement of workers

Ten healthy male mining industry employees were recruited from an open-cut coal mine site in central Queensland, Australia. Although many studies have previously used serum for biomarker analysis, industrial regulatory requirements and the need to use procedures acceptable to participants, urinary analysis was adopted. Therefore, clean catch, midstream urine samples were collected from each worker at 12 hour intervals. Pre-shift samples were collected at 18:00 hours prior to commencing an overnight 12 hour shift (PRE). Post-shift samples were collected at the end of the same shift at 06:00 hours (POST). Additional samples were collected after a 12 hour recovery period, prior to commencement of the next shift (24 hr). Samples were stored overnight at 4°C and then transported on ice to the QUT Institute of Health and Biomedical Innovation, Brisbane where they were stored at −20°C until required for analysis.

### Urea and Cortisol assays

All urine samples were thawed at room temperature and a single aliquot of each sample was sent to Queensland Medical Laboratories (Murarrie, QLD, Australia) for analysis. Urinary urea was measured by an automated kinetic assay, while creatinine levels were analysed by the Jaffe method (to standardise for dieresis). Both assays were performed using a Roche Cobas Integra 800 analyser (Roche Diagnostics, Basel, Switzerland; analytic coefficient of variation of <4% and <3% respectively). Urinary cortisol levels were measured by competitive immunoassay using a Bayer Centaur Immunoassay System (Bayer Diagnostics, Tarrytown, NY, USA: analytic coefficient of variation of <4%).

### Sample preparation for SELDI-TOF MS analysis

The remaining thawed sample was clarified by centrifugation at 1500× g for 10 minutes, aliquoted and stored at −20°C until required for ultra-filtration. Single aliquots of each sample were then thawed and pre-filtered through a 0.2 µm syringe filter (supplier) prior to ultra-filtration of 4 mL of sample at 4000× g for 40 min using Amicon Ultra-4 3000 NMWL centrifugal ultra-filtration devices (Millipore, Billerica, MA, USA). The urinary protein (retentate) was washed with 3.5 mL of milli-Q water (Millipore) and centrifuged as above to desalt the sample prior to the storage of individual aliquots at −80°C until required for analysis. A urine sample obtained from an independent male subject was identically prepared to serve as a quality control (QC).

The protein concentration of each urinary protein sample was determined in triplicate by bicinchoninic acid (BCA) protein assay (Pierce, Rockford, IL, USA) as per the manufacturer's instructions and adjusted to 0.42 mg/mL in binding buffer (10 mM sodium acetate (NaAc), pH 4.5). CM10 (weak cation exchange) ProteinChip® arrays (Bio-Rad, Hercules, CA, USA) were then pre-equilibrated twice with 50-µl of binding buffer using a ProteinChip Cassette – Compatible Bioprocessor® (Cat # C50-30011) (BioRad) and placed on a plate shaker for 5 min. Triplicate 35 µL sub-samples of urinary protein and a single QC replicate sample were applied to the surface of the CM10 arrays in a semi-randomised fashion to ensure that no replicate samples occupied a second spot on individual arrays. Since CM10 arrays have previously been found to provide good quality spectra [26], [27] from urine samples and our own (Unpublished) optimisation studies using urine samples also demonstrated good spectral reproducibility and peak number, we elected to utilise this array type for this study. The samples were allowed to bind for 1 hr on a plate shaker prior to washing each spot for 3×5-min with binding buffer, followed by 2×1 min washes with ddH_2_O. The arrays were allowed to air dry prior to, between and following 2×1 µL applications of 50% Sinapinic acid (SPA) in 50% ACN:0.5% TFA. Each array was then analysed on a PCS 4000 personal edition SELDI-TOF Mass Spectrometer (Bio-Rad).

### Data acquisition, inspection and pre-processing

The QC sample served as a quality assurance measure with the purpose of determining any batch or chip variability downstream of the data acquisition. Spectra were generated within the range of 0 to 20000 Da, matrix attenuation was set to 500 Da, focus mass was set to 5000 Da and sampling rate was set at 800 MHz, A partition of 1 of 4 was employed and each array spot received 2 warming shots at 1600-nJ and 15 data shots at 1500-nJ. A total of 530 shots per spot were acquired for analysis. Data generated from the warming shots were excluded from the averaged spectra. A calibration peptide chip was prepared using pure porcine dynorphin (2147.5 Da), bovine insulin (5733.5 Da) and bovine ubiquitin (8564.8 Da) (Bio-Rad) according to the manufacturer's instructions and run on the same day as the worker samples.

Using ProteinChip® Data Manager Software 3.0.7 (Bio-Rad), all spectra were normalised by total ion current between 2000 Da and 20000 Da. The initial spectral analysis incorporated exclusion based on Normalisation factor (Nf). Any spectra with a Nf greater than 2.5 standard deviations from the mean were excluded. Next, an external calibration equation was generated from the calibrant spectra and applied to each worker spectrum. A smoothing window of 25 points was used before fitting the baseline and filtering parameters were set to ‘on’ with an average peak width of 0.2 times the expected peak width. The spectra were collected and the peaks were clustered using Cluster wizard™ (Bio-Rad) with the following parameters unless otherwise stated; S/N = 3.0, valley depth = 3.0, centroid fraction = 0.1%, second pass option off. Individual peak clusters were accepted with m/z coefficients of variation (CV) of 0.5% or less to ensure accurate peak alignment.

Chip to chip variation was assessed by Pearson's product-moment correlations of the QC control spectra in open source *‘R’* statistical computing and graphics program, version 2.10.1 (www.r-project.org). A regression coefficient of less than r = 0.84 was set as criteria for further analysis of chip to chip variability as described previously [28]. In order to assess peak reproducibility, the CVs for individual peaks were calculated for each set of experimental replicates. Peaks with a CV below 20% were considered as sufficiently reproducible and were accepted for further analysis.

### Gel electrophoresis and protein isolation

For non-reducing SDS-PAGE, 28.5 µg (Silver staining/protein ID) or 10 µg (WB) of concentrated urinary protein per sample was prepared in 4× NuPAGE® LDS sample buffer (Invitrogen, Carlsbad, CA, USA) and loaded into NuPAGE® 4%–12% bis-tris gradient gels (Invitrogen). The samples were then electrophoresed in 1× NuPAGE® MES SDS running buffer (Invitrogen) at 200 V for 20 min at 4°C. Silver staining was performed using the SilverSNAP® Stain Kit II (Pierce) as per the manufacturer's instructions.

In order to isolate specific protein bands, a portion of an unstained duplicate gel corresponding to the band of interest was excised, placed into an eppendorf tube containing SELDI-TOF MS binding buffer and finely diced with a razor blade. The diced gel pieces were incubated at RT for 3 days in 50% ACN after which the supernatant was transferred to a separate tube and the gel pieces were then incubated in 100% ACN with shaking for a further two days. The 2 supernatants were combined and the protein-containing solution was dried under vacuum in a centrifuge. The protein pellet was resuspended in 50 mM NH_4_HCO_3_, pH 7, and a portion of the mixture was then applied to a CM10 protein chip array and subjected to SELDI-TOF MS as described above.

### Protein identification

Concentrated urinary protein (W4 and W5) were run in triplicate on fresh SDS-PAGE gels. The gels were stained with Coomassie Blue and the ∼20 kDa bands of interest were excised and then subjected to in-gel digestion. Briefly, the gel pieces were de-stained and proteins within gel pieces were reduced with 10 mM dithiothreitol, alkylated with 55 mM iodoacetamide, washed with 50 mM ammonium bicarbonate, pH 7.9 and then digested for 14 hours at 37°C with trypsin (1∶20) before being extracted into a 60% acetonitrile, 0.1% formic acid solution.. The digested protein was then subjected to reverse phase LC-MS/MS using an 1100 Series HPLC (Agilent Technologies, Boblingen, Germany) coupled with a QStar Elite Quadrupole – Time Of Flight Mass Spectrometer (Applied Biosystems, Foster City, CA, USA). The resulting peptide mass spectra were analysed using the MASCOT search algorithm against the Ludwig Non-Redundant database and subsequently validated using the Trans Proteomic Pipeline (TPP) and the Peptide Prophet tool [29], [30], [31].

### Western blot analysis

Concentrated urinary proteins samples were transferred to a NT nitrocellulose membrane (Pall Corporation, Pensacola, FL, USA) in 25 mM Tris base, 40 mM Glycine, 10% v/v Methanol, following SDS-PAGE as described above. The membrane was blocked overnight in 5% w/v skim milk powder (SMP)/TBST (100 mM Tris, 150 mM NaCl and 0.1% v/v Tween 20) at 4°C, washed for 6×5 min in TBST and incubated for 1 hr at RT with a goat anti-human endorepellin primary antibody (R&D Systems Minneapolis, MN, USA) (1∶10,000) in 5% SMP/TBST. The transfer was then washed for 6×5 min and incubated for 1 hr at RT with HRP-conjugated rabbit anti-goat secondary antibody (1∶10,000) (R&D Systems) prior to a further 6×5 min washes with TBST and detection using an ECL Plus western blot detection kit (GE Healthcare, Little Chalfont, Buckinghamshire, UK) as per the manufacturer's instructions.

### Statistical Analysis

Data are presented as mean ± standard error (SE) or standard deviation (SD) as indicated. Students *t*-test (worker physical activity characteristics) or Mann Whitney-U test (cortisol, urea and m/z 16881 data) were performed to test for group differences with significance accepted at p<0.05. Pearson correlations were used to determine the strength of association between replicate control data to assess potential chip to chip variation.

## Results

### Subject demographics

The participants comprised 10 healthy male mine workers with 4 working in maintenance (crew) and 6 in truck driving (operators) work categories. All workers performed a 12- hour overnight shift at an open- cut coal mining operation in central Queensland, Australia. The average age of participants was 38.5 years with a range from 26 to 61 years. Years of service in the mining industry ranged from 2 to 30 years with an average of 6.9 years. The average height, weight and BMI ± SD were 173 cm±6 cm, 83.70 kg±17.75 kg and 27.97 kg/m^2^±4.79 kg/m^2^, respectively. Six of the 10 workers indicated a previous history of musculoskeletal injury, with the most recent injury occurring 3- months prior to the survey.

Workers perceptions of their exposure to physical activity during work indicated that those in maintenance categories spent more time standing and walking during the shift than the operators (p<0.05) (Table 1). Importantly, only one worker, an operator (W5), reported performing physical exercise outside of work hours in the 24 hr period prior to the initial collection of urine. In terms of general physical activity the crew reported that they spent significantly less time seated and significantly more time standing and walking during the shift compared to the operators (p<0.05) (Table 1).

**Table 1:** Summary of physical exposure relative to different postures and activity.

% of shift spent	Maintenance crew	Operators (Truck drivers)
Seated	16.5%±7.8% (n = 4) *	85.9%±8.0% (n = 6)
Standing	67.2%±30.1% (n = 4) *	9.1%±5.9% (n = 5)
Walking	56.1%±32.1% (n = 3) *	8.4%±6.6% (n = 5)

* **= p<0.05.**

### Urinary urea and cortisol assays

**Figure 1:**
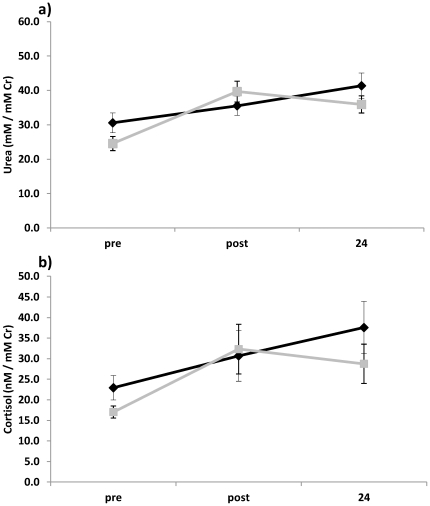
Urinary urea and cortisol levels trend toward recovery in operators but not in maintenance crew. **a**) Urinary, urea levels were determined by an automated kinetic assay (analytic coefficient of variation being <4%). **b**) Urinary cortisol levels were determined by competitive immunoassay (analytic coefficient of variation being<4%). Both urea and cortisol measurements were standardised for dieresis against urinary creatinine levels which were determined by the Jaffe method (analytic coefficient of variation being<3%).

It was established that sampling procedures for mining employees needed to be non-invasive in order to conform to workplace requirements. Given that workers were already involved in occupational urine testing, the ideal source of sampling was considered to be urine. As reported in table 2, average urinary urea levels increased significantly at both the post-shift (post) (p<0.01) and 12 hr post-shift (24 hr) (p<0.01) samples compared to pre-shift samples (pre). Similarly, urinary cortisol levels significantly increased post-shift when compared to pre-shift levels (p<0.05) and remained elevated prior to the next shift (p<0.05). Interestingly, analysis of the urea and cortisol data for each cohort indicated that although there was no significant difference in the level of urea or cortisol between crew and operators, there was a trend in the latter towards recovery. This response is similar to that expected for a circadian rhythm [18], whereas the crew did not display a trend toward recovery in urea or cortisol levels, possibly, suggesting exposure to increased physical stress (Fig. 1a & b).

**Table 2:** Urinary biomarker values for mine site employees.α

		PRE _18:00_	POST _06:00_	24 hr _18:00_
UREA	*mM/mM Cr*	26.55±1.76	38.00±2.13*	38.09±2.16*
CORTISOL	*nM/mM Cr*	19.00±1.53	31.68±4.16#	32.28±3.88#

α Values are means±SE.

* Indicates values are significantly greater than PRE value (P<0.01).

# Indicates value significantly greater than PRE value (P<0.05).

### Urinary protein analysis by SELDI-TOF MS

**Figure 2:**
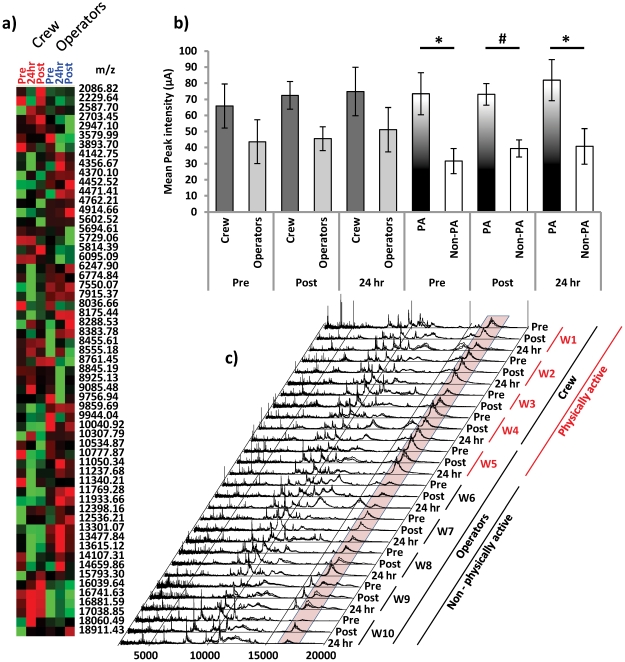
The spectral feature at m/z 16,881 is associated with physically active workers. **a**) Cluster analysis of SELDI-TOF MS data from urinary protein of mining workers. The 3 values centred around m/z 16881 constitute a major feature differentiating between crew (Red, Pre, Post & 24 hr) and operators (Blue, Pre, Post & 24 hr). Red squares indicate peak intensities above the average and green squares indicate peak intensities below the average intensity for the specified m/z value. Note block of m/z values centred on m/z 16881. **b**) Closer examination of the spectral feature m/z 16881 revealed that although the mean peak intensity appeared different between crew and operators at each time point the difference was not significant. Further examination of individual worker spectra indicated that 1 operator (W5) had a relatively high peak intensity at m/z 16881 in all three of his samples and according to information provided in the health and physical activity questionnaires W5 was the only participant to have engaged in a gym workout (resistance training) within the 24 hr period prior to sampling. Thus W5 and the crew were re-classified as the physically active (PA) cohort (n = 5) and the remaining operators were reclassified as the non-physically active (Non-PA) cohort (n = 5). The peak intensity of m/z 16881 was significantly higher in the PA workers compared to the Non-PA workers. Data is the mean peak intensity +/− SEM. Significance is given as * p<0.05 or # p<0.01 (Mann Whiney - U Test) **c**) The spectral feature centred around m/z 16881 (shaded box) generally displays a higher intensity in the physically active workers compared to workers who were less physically active. Spectral profiles for each worker are stacked replicates (n = 3 per profile).

In this study we generated SELDI-TOF MS profiles of mining worker urinary proteins to detect novel biomarkers of musculoskeletal injury, fatigue and/or physical exertion. Cluster analysis of the spectra was performed to reveal those spectral peaks which were associated with either the maintenance crew cohort, who were engaged in more physically active work, or the operator cohort, who were less physically active during the shift. This resulted in a cluster of 59 spectral peaks, of which a block of 3 peaks at m/z 16741.63, 16881.59 and 17038.85 were of particular interest since these appeared to be up-regulated in the more active group (Fig. 2a). Analysis of the peak intensities of the central spectral feature at m/z 16881 indicated that the observed difference between crew and operators was not statistically significant (Fig. 2b). However, inspection of the raw spectra revealed that worker 5 (W5), a member of the operator group, had peak intensities for the central m/z 16881 peak which were more similar to those of the crew cohort than others in his work category. Examination of the self reported out of work activities revealed that only W5 had engaged in a gym workout in the 24 hr period prior to urine sampling. Taken together these findings suggested that W5 might have been more correctly associated with the physically active maintenance workers than the less physically active operator work category. Based on this rationale we re-classified the cohorts into physically active (crew plus W5) vs non-physically active (operators without W5) for subsequent analysis. We found a significant difference in peak intensity between the physically active and non-physically active workers pre-shift (p<0.05), post shift (p<0.01) and 24 hr (p<0.05) time points (Fig. 2b). Furthermore, inspection of the raw stacked spectra for each worker clearly demonstrated the difference in peak intensity of the feature surrounding m/z 16881 between the cohorts (Fig. 2c).

### Protein isolation and identification

**Figure 3:**
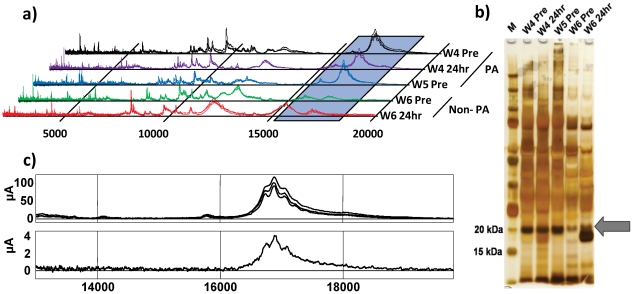
The spectral feature at m/z 16881 is a broad tri-phasic peak, visible by SDS-PAGE. **a**) The hypothesised pattern of intensity of m/z 16881 in stacked replicate spectra, expected to be observed in an SDS-PAGE gel. **b**) A band which matched the expected pattern of intensity for the feature at m/z 16881 was detected at ∼20 kDa by SDS-PAGE (**arrow)** suggesting that the bands at ∼20 kDa in the gel were the proteins which constituted m/z 16881 in the spectra. **c**) The protein at ∼20 kDa was extracted from excised bands from a non-stained replicate SDS-PAGE gel. Examination of the extracted protein by SELDI-TOF MS confirmed that the ∼20 kDa band was the feature originally detected at m/z 16881.

We then determined that it may be possible to detect this broad tri-phasic peak by silver stain following SDS-PAGE. Therefore, 2 samples were selected from a crew member (W4 Pre & W4 24 hr) and 2 samples from an operator (W6 Pre & W6 24 hr) based on maximum (W4) and minimum (W6) observed spectral peak intensities for m/z 16881 (Fig. 3a). The W5 pre-shift sample was also included as we hypothesised that the peak of interest would appear as a more intensely staining band in the W4 and W5 samples compared to the W6 samples (Fig. 3b). Indeed, we found an intensely staining band corresponding to an approximate molecular weight of ∼20 kDa in the W4 and W5 samples which did not appear as intense in the W6 samples (Fig. 3b).

In order to confirm that the intensely staining band was indeed the tri-phasic peak detected in the SELDI spectra at m/z 16881 we again performed the SDS-PAGE with the same samples in replicate gels. The ∼20 kDa band was excised, isolated and subjected to SELDI-TOF MS. We detected a tri-phasic peak at the corresponding m/z to the original analysis, although the intensity was much lower than in the original spectra (Fig. 3c). This was not unexpected since we only used a portion of the diffused protein from the excised gel piece. This data indicated that the protein band observed and excised from the SDS-PAGE gel was in fact the same protein responsible for the tri-phasic peak detected in the original SELDI-TOF mass spectra at m/z 16881.

**Figure 4:**
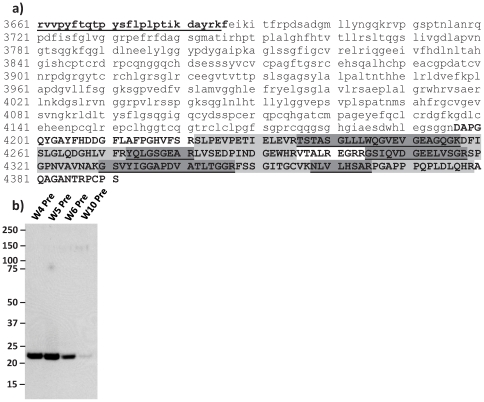
LC- MS/MS identifies the LG3 peptide of endorepellin, a C-terminal bioactive fragment of Perlecan. a) Perlecan (**underlined bold lower case**), the C terminal of Perlecan containing Endorepellin (lowercase text) and the LG3 Peptide of endorepellin (**BOLD CAPITALS**). Individual peptides identified by LC-MS/MS of tryptic in-gel digest in **LIGHT GREY** and **DARK GREY** highlights. Sequence coverage includes the LG3 peptide, however, the first 25 residues of the LG3 peptide were not detected. **b**) Western blot analysis confirmed that the ∼20 kDa protein observed by SDS-PAGE and the spectral feature at m/z 16881 are derived from endorepellin. Western Blot of worker urine samples using goat anti-human endorepellin polyclonal antibody (1∶10,000).

To identify the protein, or proteins, contained in the ∼20 kDa band and the spectral feature surrounding m/z 16881, triplicate gel pieces from 2 individual workers (W4 and W5) were excised from freshly prepared gels. The protein contained in the gel pieces was then subjected to in-gel digestion and the tryptic peptides were analysed by LC-MS/MS. The resulting peptide mass spectra were analysed using the MASCOT search algorithm. A specific fragment of the Basement Membrane Specific Heparin Sulphate Proteoglycan Core Protein – 2 (HSPG2) or Perlecan, Non-Secretory Ribonuclease were identified with high probability scores (all >54) (Table 3). The MS/MS data were then validated using the Trans Proteomic Pipeline (TPP) and the Peptide Prophet tool which resulted in a return of 30 spectra with probability scores above the calculated Minimum Probability Threshold (MPT) of 0.76, corresponding to p = 0.05. Twelve of these 30 peptides (with a MPT of >0.91, p = 0.025) corresponded to peptides which directly identified the Perlecan protein LG3 fragment (Table S1). Analysis through Protein Prophet gave a confidence value of 1.000 for both the Perlecan protein (fragment) and the Perlecan protein with the probability for these as 1.0000 and 0.9899, respectively (Table S2). Further analysis of the Perlecan sequence indicated that the peptides identified by MS/MS mapped exclusively to domain 5 of the parent protein and specifically to the C terminal LG3 peptide. Domain 5 of perlecan is also known as endorepellin which itself is a bioactive derivative of perlecan once enzymatically liberated [32], [33]. The LG3 peptide molecule spans 195 amino acids (residues 4197–4391 of the parent protein) and has a theoretical mass of 20549 Da which corresponds with the protein band observed on the gel (Fig. 3b). However, the theoretical mass did not correspond to the original m/z 16881 feature detected in the original SELDI-TOF MS data. In order to further characterise the sequence coverage of our MS/MS data and to account for this apparent discrepancy, we performed an *in silico* trypsin digest of the LG3 peptide using the Peptide Mass tool on ExPasy Proteomics Server. We found that the peptides identified by LC-MS/MS matched the *in silico* generated tryptic fragments and collectively resulted in 95.9% sequence coverage of the putative LG3 peptide (Fig. 4a and Table S3). Interestingly, our MS/MS data did not identify any sequence from the first 25 residues of the LG3 peptide. The sequence coverage of our experimental data spanned 158 amino acids incorporating residues 26 to 183 of the parent LG3 peptide or 4222–4379 of the perlecan parent sequence and had a theoretical mass of 16641 Da, which more closely corresponded to the m/z of our original SELDI-TOF MS experimental data.

**Table 3:** The ∼20 kDa band excised from the SDS-PAGE gel is a fragment of perlecan.

Sample	Acc. No.	Proteins	Mass (kDa)	Score α	Matching peptides
W4 Pre	P98160	Basement membrane-specific heparan sulfate proteoglycan core protein	479.3	329	10
	P10153	Non-secretory ribonuclease	18.8	180	4
W4 Pre	P10153	Non-secretory ribonuclease	18.8	140	4
	Q59EG0	Basement membrane-specific heparan sulfate proteoglycan core protein (Fragment)	249.9	130	5
W4 Pre	P10153	Non-secretory ribonuclease	18.8	102	3
	Q59EG0	Basement membrane-specific heparan sulfate proteoglycan core protein (Fragment)	249.9	71	3
	P98160	Basement membrane-specific heparan sulfate proteoglycan core protein	479.2	67	3
W5 Pre	Q59EG0	Basement membrane-specific heparan sulfate proteoglycan core protein (Fragment)	249.9	211	7
	P98160	Basement membrane-specific heparan sulfate proteoglycan core protein	479.2	204	7
	P10153	Non-secretory ribonuclease	18.8	123	4
W5 Pre	Q59EG0	Basement membrane-specific heparan sulfate proteoglycan core protein (Fragment)	249.9	169	6
	P98160	Basement membrane-specific heparan sulfate proteoglycan core protein	479.2	162	6
	P10153	Non-secretory ribonuclease	18.8	53	2
W5 Pre	P98160	Basement membrane-specific heparan sulfate proteoglycan core protein	479.2	275	9
	P10153	Non-secretory ribonuclease	18.8	133	4

Mascot search results.

α Ions score is −10*log(P), where P is the probability that the observed match is a random event. Individual ion scores >52 indicate identity of extensive homology (p<0.05).

In order to further confirm that the protein band observed at ∼20 kDa was indeed derived from perlecan/endorepellin we subjected 4 worker urine samples which exhibited high (W4 Pre and W5 Pre) and low (W6 Pre and W10 Pre) intensity SELDI-TOF MS peaks at m/z 16881 to western blot analysis using a polyclonal antibody raised against full length endorepellin. A strong immunoreactive band was observed which corresponded to the ∼20 kDa band observed in the silver-stain of worker urinary protein, indicating that the band was derived from endorepellin (Fig. 4b). Moreover, the intensity of the bands was consistent with the difference in intensity of the peaks at m/z 16881 observed in the original SELDI-TOF MS data. (Unfortunately, we were unable to perform additional analysis of the samples from the current study due to insufficient sample). Additional analysis of urine samples provided *ad libitum* from an additional 6 workers from a separate study revealed variation in LG3 expression over a shift, suggesting acute expression and thus coordination between sample collection and the inducing activity may be important (Fig S1, S2, S3). The strongest and most consistent expression was detected in single maintenance worker (Fig S2), According to self report, none of the workers from the separate study had engaged in intensive physical activity prior to the sampling period. In addition, the Non Secretory Ribonuclease, which was co-identified by mass spectrometry, exhibited an increasing but different pattern of expression across an individual time course series compared to the LG3 peptide (Fig S4).

## Discussion

Despite increased automation of tasks with high risk of injury, many tasks are unable to be redesigned and this is demonstrated in task analyses of maintenance workers in mining which are described as a combination of interspersed intensive resistance exertion and light to moderate aerobic physical activity [34]. Furthermore, the overall length of exposure to such exertion is a contributing factor to injury and a factor potentially responsible for the persistent increase in urinary urea and cortisol levels observed in the maintenance workers in this study. While there is prior evidence for increased urea excretion in response to acute resistance exercise [35], altered levels may also be associated with activities of higher intensity and longer duration [36], [37], or dietary amino acid catabolism [38]. As such, caution in the interpretation of physical activity levels based on urea production alone is imperative. Similarly, caution must be applied in the interpretation of cortisol responses, since cortisol secretion exhibits a circadian rhythm where levels peak in the morning and nadir at night [18]. In our study, cortisol and urea levels were measured in samples collected before and after the second night shift of the roster. Thus it is possible that the differences between pre- and post-shift values were due to normal circadian variation in cortisol secretion. Indeed, this may explain the observed trend toward recovery in cortisol levels for the operators in the 24 hr post-shift sample (Fig. 1). However, there was no observed recovery in cortisol levels for the maintenance crew, which suggests there is a difference between the 2 cohorts in terms of stressors.

It has been hypothesised that long hours and extended working periods may not enable sufficient recovery from work and may contribute to health problems in workers [39]. While our data may support this hypothesis, a study by Garde *et al.* (2006) found no difference in cortisol levels between employees working extended hours (12 hour work days and longer rosters) compared to those with regular (8 hr) work schedules [40]. However, this discrepancy may be explained by sampling conditions, since the authors measured cortisol in saliva samples over day shifts, while our urine samples were collected spanning the second night shift of the roster. Indeed, while it is possible for some workers to develop a tolerance to shift work and adapt their cortisol functions accordingly, evidence suggests that this does not occur until the fifth night of the roster [18]. Conversely, other studies have observed lower cortisol values when sampled in the morning following night work [41], [42], rather than increased values as we observed in the crew cohort. These studies involved health care workers who may not be subjected to the same level of physical activity as those in mining activities as in this study. When considered together, our cortisol and urea data support the hypothesis that there is a lack of recovery from work in the maintenance crew compared to the more sedentary operator cohort.

The analysis of worker urinary protein by SELDI-TOF MS offered an additional means by which to discover novel biomarkers of musculoskeletal injury or of exposure to associated risk factors, such as prolonged physical activity. The initial cluster analysis of our SELDI-TOF MS data revealed that a number of m/z peaks were associated with either the maintenance or driver cohorts. The most striking of these was a tri-phasic cluster with a central m/z of 16881 (Fig. 2), which we were subsequently able to visualise by SDS-PAGE (Fig. 3) and identify as containing Non-Secretory Ribonuclease (NSR) and the LG3 peptide of endorepellin using LS-MS/MS (Fig. 4, tables S1, S2, S3 and Fig S4). While the NSR, otherwise known as Eosinophil Derived Neurotoxin (EDN) was not examined further in this study we did compare its expression to that of the LG3 in a separate study and found it to increase across a time course series in a single worker (Fig S4). While the significance (if any) of this expression pattern remains unknown it is possible that it may also be associated with physical activity level but exhibits a less acute expression than the LG3 peptide. Urinary expression of the NSR/EDN has been associated with eosinophil degranulation and allergic conditions such as atopic dermatitis and asthma [43], [44]. The NSR/EDN is also expressed in macrophages and is involved in inflammatory processes and the innate immune response inflammatory [45]. Therefore, it may be interesting in longer term controlled studies to further examine the release profile of the NSR/EDN following different levels of physical activity intense physical activity can illicit inflammatory effects.

To the best of our knowledge this is the first study to demonstrate that physically active workers have higher urinary levels of the LG3 peptide of endorepellin. The LG3 peptide is the C-terminal bioactive proteolytic fragment of endorepellin, which is itself an 80 kDa bioactive C-terminal protein of perlecan [32]. Interestingly, perlecan is a major extracellular matrix constituent of all basement membranes and significantly, articular cartilage, and neuromuscular junctions [46], [47], [48]. In 2003, Mongiat *et al.* hypothesised that endorepellin might be released from articular cartilage during remodelling or inflammation [32]. Given that during intense physical activity/exercise, both muscle tissue [49], [50], [51] and articular cartilage [52] are subjected to forces capable of inducing inflammation and tissue remodelling processes, the appearance of the LG3 peptide of endorepellin in the urine of physically active mining workers, as described in this study, supports the hypothesis proposed by Mongiat *et al.* (2003).

While perlecan is known to have pro-angiogenic functions *in vivo*, its C-terminal bioactive fragment, endorepellin, is an inhibitor of angiogenesis [32]. Furthermore, the LG3 peptide, originally discovered in the urine of end-stage renal failure patients [53], has been characterised as the anti-angiogenic moiety of endorepellin [32], [54]. More recently, Chang *et al. (2008)* reported that conditioned media from the malignant breast cancer cell line, Hs578T, was found to have lower levels of LG3 peptide compared to the non-tumour breast cell line Hs578Bst [55]. Moreover, these authors found that breast cancer patients (n = 6) had lower levels of circulating LG3 peptide compared to healthy volunteers (n = 6) and therefore proposed that low circulating LG3 peptide might be a potential biomarker of breast cancer [55]. It is important to note here that our data may provide a different interpretation to the results reported by Chang *et al.* (2008), in that it is possible that the healthy volunteers recruited for their study were simply more active than the cancer patients prior to sample collection [55]. Moreover, it is unlikely that one will observe lower levels of circulating LG3 in cancer patients if their tumour is secreting lower levels than the rest of the body, as suggested by the authors finding that breast cancer cells produced lower levels of LG3 *in vitro*. Chang *et al.* (2008) also noted that the only other studies which had described altered LG3 peptide levels were associated with urine analysis of patients with end stage renal failure [53] and in amniotic fluid during premature foetal membrane rupture in pregnant women [56]. Our data provides evidence for a potential additional explanation for the appearance of the LG3 peptide in urine. Clearly the exact nature and circumstances surrounding the alteration to circulating and urinary LG3 peptide or endorepellin requires further investigation.

In further support of our data, the LG3 peptide is known to be proteolytically released from endorepellin and perlecan by both bone morphogenetic protein–1 (BMP-1)/Tolloid like Metalloprotease [33] and caspase-3 mediated Cathepsin-L mechanisms [57]. Recent studies have demonstrated that BMP-1 is released from cartilage explants following exposure to mechanical injury or to either of the inflammatory cytokines tumour necrosis factor-α (TNF-α) or interleukin-1β (IL-1β) [58]. Both of these cytokines are found in rheumatoid and osteoarthritic synovial fluid [59] and have both been shown to stimulate the expression of cathepsins in endothelial cells, macrophages and smooth muscle cells [60]. Thus, the mediators of two of the known mechanisms of LG3 peptide liberation are present and active in the articular cartilage/synovial environment and/or musculoskeletal tissues generally. Moreover, eccentric exercise is known to induce inflammatory responses such as leukocyte accumulation and up regulation of cytokines in exercised muscles [15], [61]. When considered together, these data provide an additional potential source and explanation for the increase in LG3 peptide in the urine of physically active mining workers.

The LG3 peptide/endorepellin is among a number of proteolytic fragments from articular cartilage and basement membrane proteins which have anti-angiogenic/angiostatic effects. These include endostatin, tumstatin and arrestin, all C-terminal peptides derived from collagen XVIII and the α3 and α2 chains of collagen IV respectively. In support of our data, treadmill running exercise has been shown to transiently increase circulating levels of endostatin in healthy male subjects relative to individual levels of oxygen consumption [62], suggesting that endostatin reflects the level of physical exertion. Thus, it would be very interesting to ascertain if exposure to intensive physical activity also induces the release of either tumstatin or arresten peptides. Our results have shown that the LG3 peptide is elevated in the urine of physically active workers, and that a number of anti-angiogenic peptides are known to be released from matrix proteins present in articular cartilage and/or in response to inflammatory cues or exercise. As such, we would like to propose our own “provocative hypothesis” in the spirit of that postulated by Mongiat *et al.* (2003) [32]. It is possible that the physical activity induced release of LG3 peptide/endorepellin, and other similar anti-angiogenic fragments of ECM components, may be one of the physiological mechanisms for the well documented but still not clearly understood link between physical activity and reduced cancer risk or improved survival from cancer [63], [64], [65]. Indeed, recombinant human endorepellin administration to the peritoneal cavity of nude mice or C57BL/6 mice inoculated with A431 human squamous cell carcinoma cells or Lewis lung carcinoma, respectively, restricted tumour development by specific disruption of tumour neovasculature. This resulted in tumour hypoxia, reduced proliferation, metabolism and increased tumour cell apoptosis. In addition, endostatin, tustatin and arresten have all been shown to inhibit tumour development in various model systems [66], [67], [68], [69]. While our proposed hypothesis remains to be tested, it is also important to address a number of limitations in our study. Firstly, the association between urinary LG3 peptide and physically active mining workers was observed in a small sample population and is therefore quite preliminary in nature and requires further validation in controlled laboratory settings with larger cohorts. Secondly, that the level of intensity and duration of physical activity required for LG3 peptide/endorepellin release remains to be established. This is most achievable within a laboratory environment as stated above. These studies are required in order to fully appreciate if urinary LG3 peptide levels reflect pathological injury or (as we have speculated in the above hypothesis) a therapeutic level of activity.

In conclusion, we have for the first time, described a potential association between urinary LG3 peptide levels and physical activity in a cohort of mining workers. Thus, we propose that urinary LG3 peptide may serve as a biomarker of physical activity, a known risk factor when inappropriately prescribed for musculoskeletal injury. Such biomarkers could be utilised in musculoskeletal injury risk assessment and management in the heavy industrial and sport sectors in order to reduce the risk and enhance interventions to prevent musculoskeletal injury. Moreover, due to the well established anti-angiogenic/anti-tumorigenic activity of LG3/endorepellin, we also propose that physical activity induced release of LG3/endorepellin may be a possible biological mechanism explaining the relationship between cancer risk/survival and physical activity.

## Supporting Information

Figure S1 **Normalised LG3 expression in a time course of urine samples from Worker 12 (maintenance worker).** a) Dot blot using the same sample as prepared for SDS-PAGE to allow normalisation for loading. b) Western blot for the LG3 peptide. c) Normalised densitometry data relative to the LG3 expression in an unrelated sample used as a normalisation standard for each subsequent western blot (as indicated in Fig S1, S2, S3). **Dot Blot (loading control) and Densitometry of Supplementary Western Blots** **Method**: SDS-PAGE Samples were taken out of the −80°C and placed on ice. 1.16 µg of total protein was prepared in separate tubes to a total volume of 28 µL for each sample 1. The content in each tube was mixed and pulse centrifuged. 2. 4 µL of each sample was transferred to clean eppendorf tubes for dot blot analysis (evaluation of loading). 3. 8 µL of loading buffer was added to the remaining 24 µL of samples. 4. The content in each tube was mixed and pulse centrifuged. 5. The samples were loaded into a 15 well NuPAGE Tris HCL 4–12% gradient gels. 6. The samples were electrophoresed in NuPAGE MES buffer at 200 V constant for 35 minutes at 4°C. 7. Following electrophoresis the protein was transferred to a nitrocellulose membrane using Semi-dry Transfer for 90 minutes at 45 mAmps/gel Western Blotting Following protein transfer the nitrocellulose membranes were blocked in 5% skim milk powder in Tris buffered saline/0.1% Tween 20 (TBST) for 1 hour at room temperature (RT) on a shaker and protected from contaminants with aluminium foil. 1. The blocked membrane was incubated with either a goat anti-human Endorepellin polyclonal antibody as primary (1∶10,000 dilution) or a rabbit anti-Eosinophil derived neurotoxin polyclonal antibody as primary (1∶2,500 dilution) for 1 hour at RT on a shaker. 2. The membrane was washed in TBST for 6×5 minutes at RT on a shaker. 3. The membrane was incubated in a either a 1∶10,000 dilution of rabbit anti-goat IgG HRP-conjugated secondary antibody or a 1∶5000 dilution of donkey anti-rabbit IgG HRP-conjugated secondary antibody as appropriate in TBST for 30 minutes at RT on a shaker. 4. The secondary antibody was removed and the membrane was washed in TBST for 6×5 minutes at RT on a shaker. 5. Each membrane and strip was transferred to a clean white weighing tray and incubated in Amersham™ ECL™ Prime western blot detection reagent (GE Healthcare Ltd, Little Chalfont, Buckinghamshire, UK) as per manufacturer's instructions. 6. Chemiluminescence was captured by exposure of the membrane to X-Ray film (Fujifilm, Tokyo, Japan) and subsequently developed using an Agfa automated film developer CP-1000 (Mortsel, Belgium). Dot Blot 1. 2 µL of each sample (prepared as above) was transferred to a nitrocellulose membrane strip and allowed to air dry. 2. To visualise the protein spots the membrane was reversibly stained using MemCode™ Reversible Protein Stain Kit (Peirce, Rockford, IL, USA) as per manufacturer's instructions. Briefly, **A. Stain** 1. The nitrocellulose membrane containing the proteins was rinsed with milliQ water and quickly decanted. 2. 1 mL of MemCode™ Reversible Protein Stain was added to the nitrocellulose membrane. 3. The membrane was placed on a shaker at room temperature for 30 seconds (Stained proteins appear as turquoise-blue bands). **B. Destain (remove background)** 1. 1 mL of MemCode™ Destain Reagent was added to the membrane and removed after a few seconds. This step was repeated two additional times. 2. 1 mL of the Destain Reagent was added to the membrane and agitated for 5 minutes on a shaker. 3. The membrane was rinsed four times by adding milliQ water to the tray and decanting after a few seconds. 4. The membrane was washed with milliQ water for 5 minutes on a shaker with agitation. Densitometry 1. A digital image of the dot blot was obtained using gel doc or of western blot films using a flatbed scanner. 2. Densitometry was performed using Image J (NIH).
(TIF)

Figure S2 **Normalised LG3 expression in a time course of urine samples from Workers 13 & 14 (maintenance workers).** a) Dot blot using the same sample as prepared for SDS-PAGE to allow normalisation for loading. b) Western blot for the LG3 peptide. c) Normalised densitometry data relative to the LG3 expression in an unrelated sample used as a normalisation standard.
(TIF)

Figure S3 **Normalised LG3 expression in a time course of urine samples from Workers 15, 16 & 18 (operators).** a) Dot blot using the same sample as prepared for SDS-PAGE to allow normalisation for loading. b) Western blot for the LG3 peptide. c) Normalised densitometry data relative to the LG3 expression in an unrelated sample used as a normalisation standard.
(TIF)

Figure S4 **Comparison of Normalised LG3 and Non Secretory Ribonuclease (NSR) expression in a time course of urine samples from Worker 13.** a) Dot blot using the same samples as prepared for SDS-PAGE to allow normalisation for loading. Western blot for b) the LG3 peptide or c) NSR. Normalised densitometry data for d) LG3 expression or e) NSR expression. The data in d & e are presented as relative to an unrelated sample derived from a physically active participant in a separate study (V15 BL2). Both the LG3 peptide and the NSR were identified by mass spectrometry of an in gel digest of the 20 kDa band observed and excised from an SDS-PAGE gel . The data from this sample provides additional data indicating that the NSR and the LG3 are co-identified in the same 20 KDa band. Recombinant NSR served as a positive control for NSR expression.
(TIF)

Table S1 **Peptides from all samples validated through the TPP – these 4 peptide sequences, out of the 10 identified from MS/MS spectral data, were validated with greater than 95% confidence.** The remaining 6 peptides either had a MASCOT ion score below 57 or were lower than the 95% confidence interval as calculated by PeptideProphet.
(DOC)

Table S2 **Analysis through Protein Prophet gave a confidence value of 1.000 for both the Perlecan protein (fragment) and the Perlecan protein with the probability for these as 1.0000 and 0.9899, respectively.**
(DOC)

Table S3 ***In silico*** **trypsin digest was performed on the LG3 peptide using the PeptideMass tool on the ExPASy Proteomics Server.** The following options were selected: cysteines treated with iodoacetomide; methionines oxidized with [M+H]^+^; monoisotopic peptides; no allowed missed cleavage; and peptides larger than 500 Da. The MS/MS data (indicated by the stars) fits with the data generated from the computer driven model of enzymatic-digestion. In addition, a BLAST search was performed on the LG3 peptide sequence identified and it was found that no other known protein/peptide shares sequence homology above 88% coverage.
(DOC)
