# Supplementary figures and images for: A Fragment of the LG3 Peptide of Endorepellin Is Present in the Urine of Physically Active Mining Workers: A Potential Marker of Physical Activity
Source: PLoS One. 2012 Mar 23;7(3):e33714. doi: 10.1371/journal.pone.0033714 (PMC3311645; doi:10.1371/journal.pone.0033714)

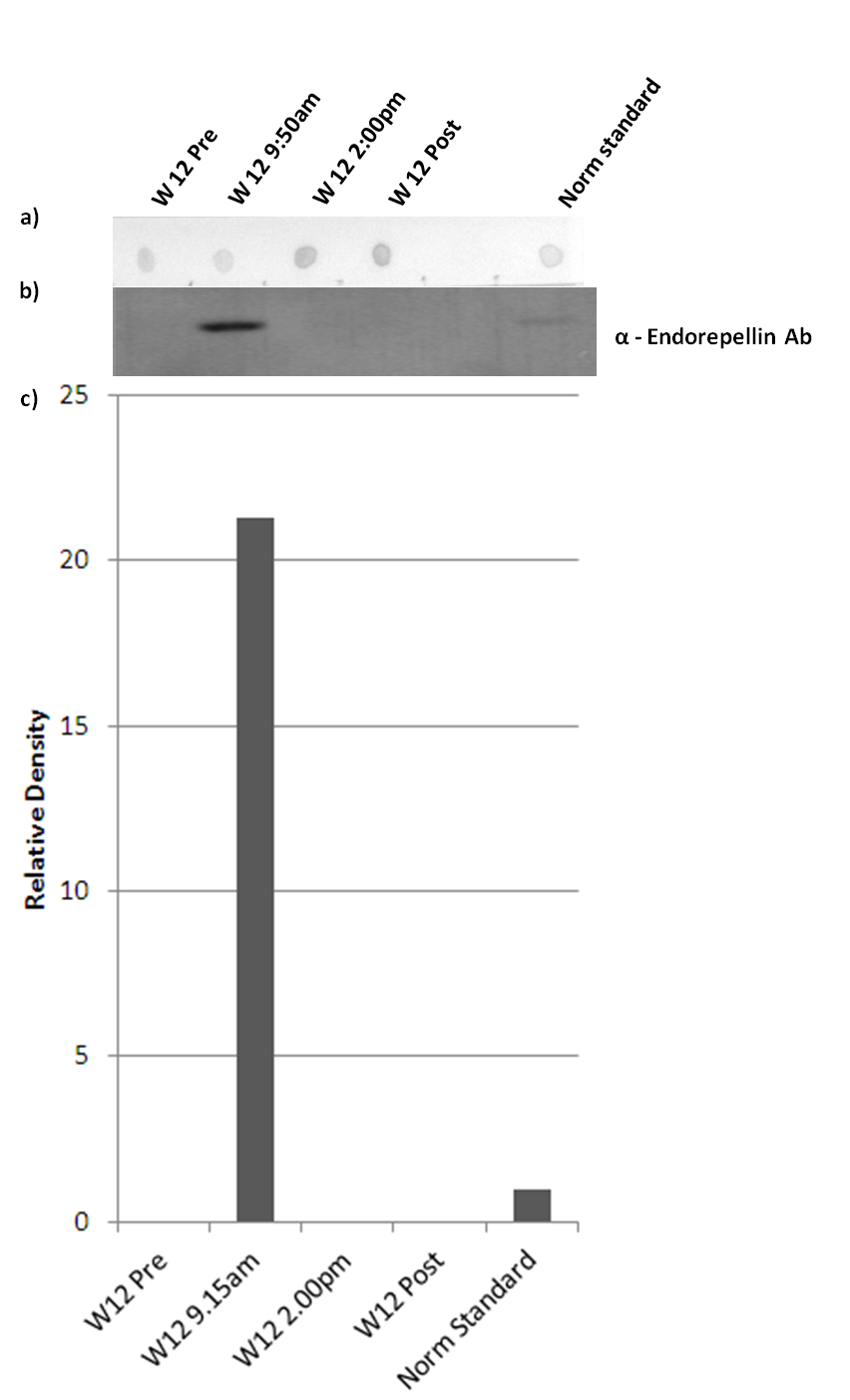

Supplement: Figure S1 — Normalised LG3 expression in a time course of urine samples from Worker 12 (maintenance worker). a) Dot blot using the same sample as prepared for SDS-PAGE to allow normalisation for loading. b) Western blot for the LG3 peptide. c) Normalised densitometry data relative to the LG3 expression in an unrelated sample used as a normalisation standard for each subsequent western blot (as indicated in Fig S1, S2, S3). Dot Blot (loading control) and Densitometry of Supplementary Western Blots Method: SDS-PAGE Samples were taken out of the −80°C and placed on ice. 1.16 µg of total protein was prepared in separate tubes to a total volume of 28 µL for each sample 1. The content in each tube was mixed and pulse centrifuged. 2. 4 µL of each sample was transferred to clean eppendorf tubes for dot blot analysis (evaluation of loading). 3. 8 µL of loading buffer was added to the remaining 24 µL of samples. 4. The content in each tube was mixed and pulse centrifuged. 5. The samples were loaded into a 15 well NuPAGE Tris HCL 4–12% gradient gels. 6. The samples were electrophoresed in NuPAGE MES buffer at 200 V constant for 35 minutes at 4°C. 7. Following electrophoresis the protein was transferred to a nitrocellulose membrane using Semi-dry Transfer for 90 minutes at 45 mAmps/gel Western Blotting Following protein transfer the nitrocellulose membranes were blocked in 5% skim milk powder in Tris buffered saline/0.1% Tween 20 (TBST) for 1 hour at room temperature (RT) on a shaker and protected from contaminants with aluminium foil. 1. The blocked membrane was incubated with either a goat anti-human Endorepellin polyclonal antibody as primary (1∶10,000 dilution) or a rabbit anti-Eosinophil derived neurotoxin polyclonal antibody as primary (1∶2,500 dilution) for 1 hour at RT on a shaker. 2. The membrane was washed in TBST for 6×5 minutes at RT on a shaker. 3. The membrane was incubated in a either a 1∶10,000 dilution of rabbit anti-goat IgG HRP-conjugated secondary antibody or a 1∶5000 d [file pone.0033714.s001.tif]

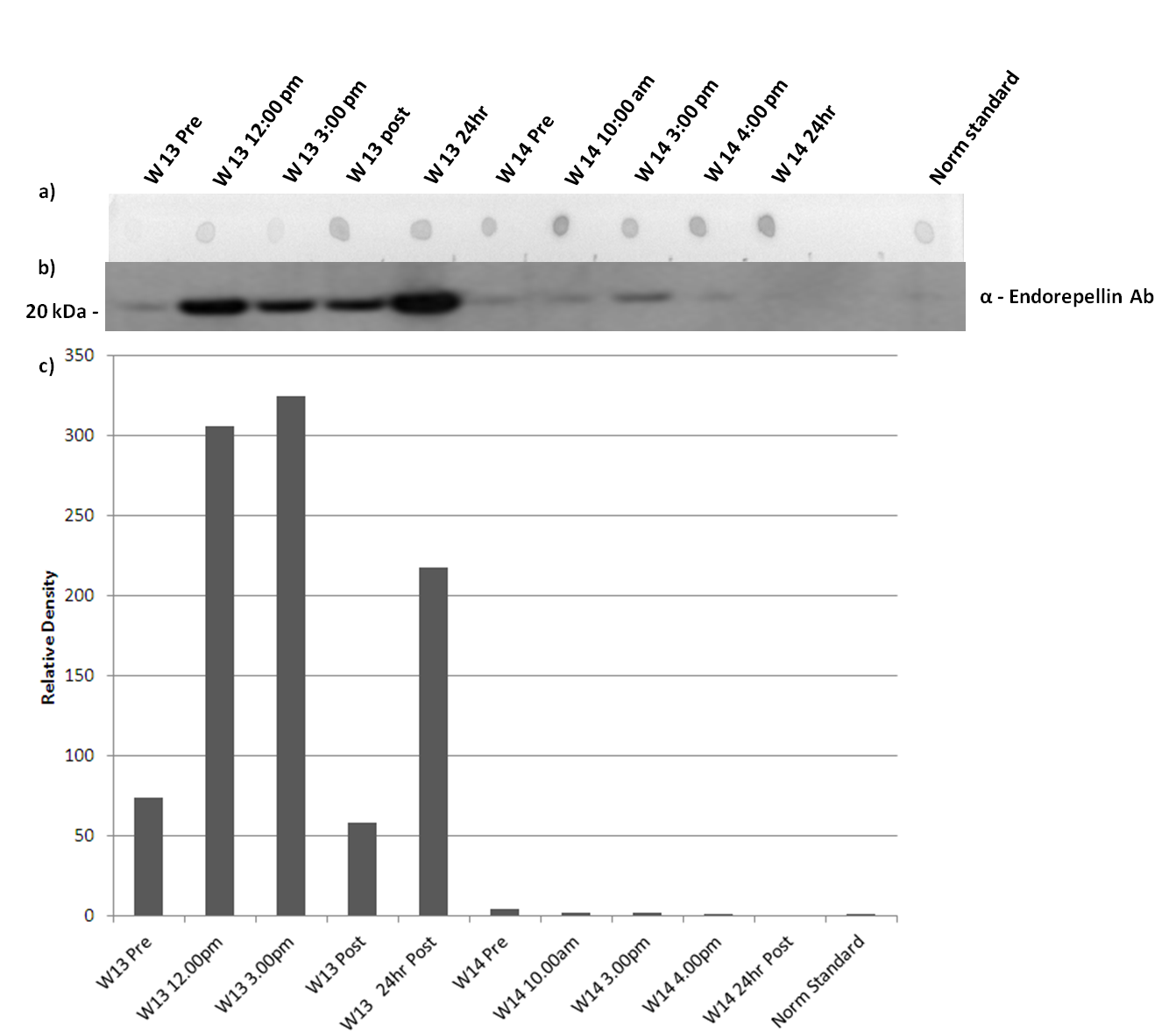

Supplement: Figure S2 — Normalised LG3 expression in a time course of urine samples from Workers 13 & 14 (maintenance workers). a) Dot blot using the same sample as prepared for SDS-PAGE to allow normalisation for loading. b) Western blot for the LG3 peptide. c) Normalised densitometry data relative to the LG3 expression in an unrelated sample used as a normalisation standard. (TIF) [file pone.0033714.s002.tif]

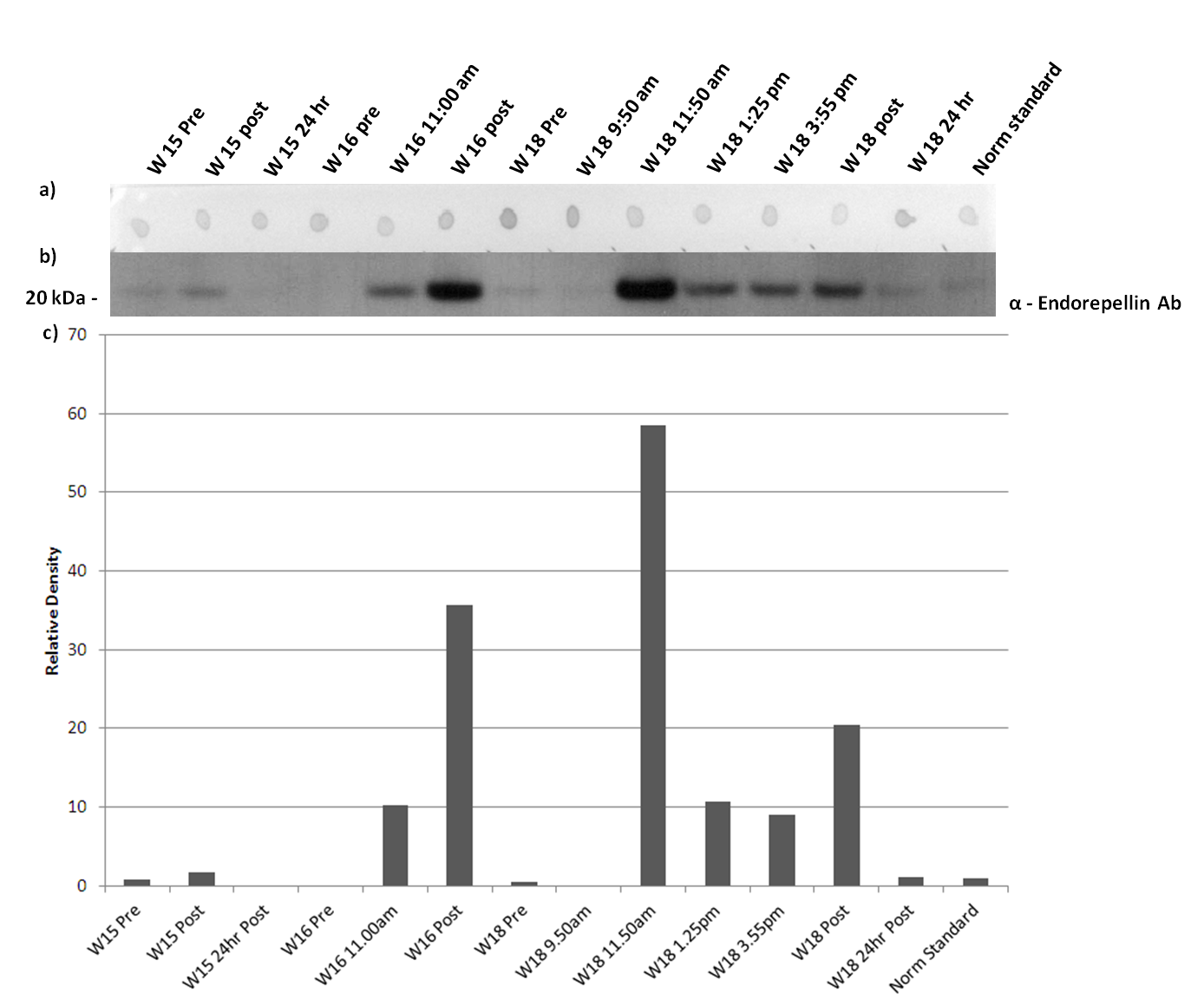

Supplement: Figure S3 — Normalised LG3 expression in a time course of urine samples from Workers 15, 16 & 18 (operators). a) Dot blot using the same sample as prepared for SDS-PAGE to allow normalisation for loading. b) Western blot for the LG3 peptide. c) Normalised densitometry data relative to the LG3 expression in an unrelated sample used as a normalisation standard. (TIF) [file pone.0033714.s003.tif]

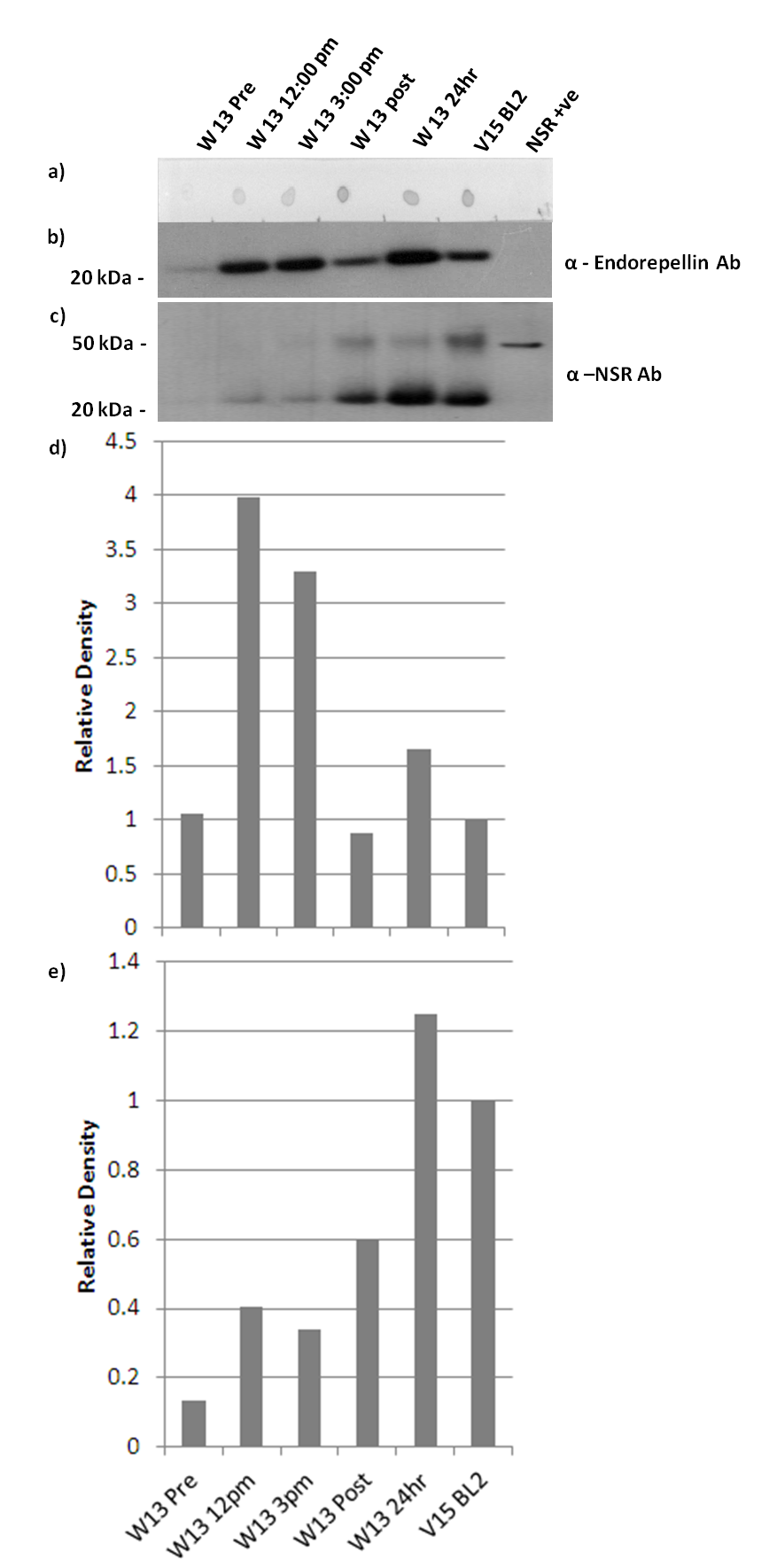

Supplement: Figure S4 — Comparison of Normalised LG3 and Non Secretory Ribonuclease (NSR) expression in a time course of urine samples from Worker 13. a) Dot blot using the same samples as prepared for SDS-PAGE to allow normalisation for loading. Western blot for b) the LG3 peptide or c) NSR. Normalised densitometry data for d) LG3 expression or e) NSR expression. The data in d & e are presented as relative to an unrelated sample derived from a physically active participant in a separate study (V15 BL2). Both the LG3 peptide and the NSR were identified by mass spectrometry of an in gel digest of the 20 kDa band observed and excised from an SDS-PAGE gel . The data from this sample provides additional data indicating that the NSR and the LG3 are co-identified in the same 20 KDa band. Recombinant NSR served as a positive control for NSR expression. (TIF) [file pone.0033714.s004.tif]
