# Supplementary material for: A Fragment of the LG3 Peptide of Endorepellin Is Present in the Urine of Physically Active Mining Workers: A Potential Marker of Physical Activity
Source: PLoS One. 2012 Mar 23;7(3):e33714. doi: 10.1371/journal.pone.0033714 (PMC3311645; doi:10.1371/journal.pone.0033714)
Supplement: Table S1 — Peptides from all samples validated through the TPP – these 4 peptide sequences, out of the 10 identified from MS/MS spectral data, were validated with greater than 95% confidence. The remaining 6 peptides either had a MASCOT ion score below 57 or were lower than the 95% confidence interval as calculated by PeptideProphet. (DOC) [file pone.0033714.s005.doc]

**Supplementary Table 1. Peptide prophet probability scores for 4 highest confidence peptides of HSPG2.**

| **Peptide**  **Prophet Probability** | **Ions** | **Peptide** | **Neutral Peptide Mass** | **Charge** | **m/z** | **pI** | **Ion Score** | **Protein** | **Acc No** |
| --- | --- | --- | --- | --- | --- | --- | --- | --- | --- |
| 1 | 9/20 | R.SPGPNVAVNAK.G | 1052.5614 | 2 | 527.288 | 8.47 | 73.87 | membrane-specific heparan sulfate proteoglycan core protein Tax_Id=9606 [Homo sapiens] | P98160 |
| 1 | 9/20 | R.SPGPNVAVNAK.G | 1052.5614 | 2 | 527.288 | 8.47 | 61.05 | membrane-specific heparan sulfate proteoglycan core protein Tax_Id=9606 [Homo sapiens] | P98160 |
| 1 | 9/20 | R.SPGPNVAVNAK.G | 1052.5614 | 2 | 527.288 | 8.47 | 67.48 | membrane-specific heparan sulfate proteoglycan core protein Tax_Id=9606 [Homo sapiens] | P98160 |
| 0.9999 | 9/34 | K.GSVYIGGAPDVATLTGGR.F | 1689.8686 | 2 | 845.9416 | 5.84 | 60.93 | membrane-specific heparan sulfate proteoglycan core protein Tax_Id=9606 [Homo sapiens] | P98160 |
| 0.9999 | 9/34 | K.GSVYIGGAPDVATLTGGR.F | 1689.8686 | 2 | 845.9416 | 5.84 | 69.98 | membrane-specific heparan sulfate proteoglycan core protein Tax_Id=9606 [Homo sapiens] | P98160 |
| 0.9869 | 9/26 | R.GSIQVDGEELVSGR.S | 1444.7158 | 2 | 723.3652 | 4.14 | 85.58* | membrane-specific heparan sulfate proteoglycan core protein Tax_Id=9606 [Homo sapiens] | P98160 |
| 0.9868 | 8/26 | R.GSIQVDGEELVSGR.S | 1444.7158 | 2 | 723.3652 | 4.14 | 71.63* | membrane-specific heparan sulfate proteoglycan core protein Tax_Id=9606 [Homo sapiens] | P98160 |
| 0.9836 | 7/26 | R.GSIQVDGEELVSGR.S | 1444.7158 | 2 | 723.3652 | 4.14 | 63.28* | membrane-specific heparan sulfate proteoglycan core protein Tax_Id=9606 [Homo sapiens] | P98160 |
| 0.9701 | 8/26 | R.GSIQVDGEELVSGR.S | 1444.7158 | 2 | 723.3652 | 4.14 | 68.07* | membrane-specific heparan sulfate proteoglycan core protein Tax_Id=9606 [Homo sapiens] | P98160 |
| 0.9651 | 7/26 | R.GSIQVDGEELVSGR.S | 1444.7158 | 2 | 723.3652 | 4.14 | 57.80* | membrane-specific heparan sulfate proteoglycan core protein Tax_Id=9606 [Homo sapiens] | P98160 |
| 0.9194 | 7/26 | R.GSIQVDGEELVSGR.S | 1444.7158 | 2 | 723.3652 | 4.14 | 54.02* | membrane-specific heparan sulfate proteoglycan core protein Tax_Id=9606 [Homo sapiens] | P98160 |
| 0.9999 | 13/34 | K.GNVYIGGAPDVATLTGGR.F | 1716.8795 | 2 | 859.447 | 5.84 | 63.29 | membrane-specific heparan sulfate proteoglycan core protein variant (Fragment) Tax_Id=9606 [Homo sapiens] | Q59EG0 |

Peptides from all samples validated through the TPP – these 4 peptide sequences, out of the 10 identified from MS/MS spectral data, were validated with greater than 95% confidence. The remaining 6 peptides either had a MASCOT ion score below 57 or were lower than the 95% confidence interval as calculated by PeptideProphet.
