# Supplementary material for: A Fragment of the LG3 Peptide of Endorepellin Is Present in the Urine of Physically Active Mining Workers: A Potential Marker of Physical Activity
Source: PLoS One. 2012 Mar 23;7(3):e33714. doi: 10.1371/journal.pone.0033714 (PMC3311645; doi:10.1371/journal.pone.0033714)
Supplement: Table S2 — Analysis through Protein Prophet gave a confidence value of 1.000 for both the Perlecan protein (fragment) and the Perlecan protein with the probability for these as 1.0000 and 0.9899, respectively. (DOC) [file pone.0033714.s006.doc]

**Supplementary Table 2**. Protein Prophet Probability scores

| **Protein** | **Probability** | **Confidence** | **coverage (%)** | **No. unique peps** | **No. indep spectra** | **share of spectrum id's (%)** |
| --- | --- | --- | --- | --- | --- | --- |
| membrane-specific heparan sulfate proteoglycan core protein variant (Fragment) | 1 | 1 | 2.6 | 4 | 18 | 6.14 |
| membrane-specific heparan sulfate proteoglycan core protein | 0.9899 | 1 | 1.5 | 1 | 4 | 7.02 |

Analysis through Protein Prophet gave a confidence value of 1.000 for both the Perlecan protein (fragment) and the Perlecan protein with the probability for these as 1.0000 and 0.9899, respectively.
