# Supplementary material for: A Fragment of the LG3 Peptide of Endorepellin Is Present in the Urine of Physically Active Mining Workers: A Potential Marker of Physical Activity
Source: PLoS One. 2012 Mar 23;7(3):e33714. doi: 10.1371/journal.pone.0033714 (PMC3311645; doi:10.1371/journal.pone.0033714)
Supplement: Table S3 — In silico trypsin digest was performed on the LG3 peptide using the PeptideMass tool on the ExPASy Proteomics Server. The following options were selected: cysteines treated with iodoacetomide; methionines oxidized with [M+H]+; monoisotopic peptides; no allowed missed cleavage; and peptides larger than 500 Da. The MS/MS data (indicated by the stars) fits with the data generated from the computer driven model of enzymatic-digestion. In addition, a BLAST search was performed on the LG3 peptide sequence identified and it was found that no other known protein/peptide shares sequence homology above 88% coverage. (DOC) [file pone.0033714.s007.doc]

**Supplementary Table 3. The peptides identified by MS/MS match an *in silico* digest of the LG3 peptide.**


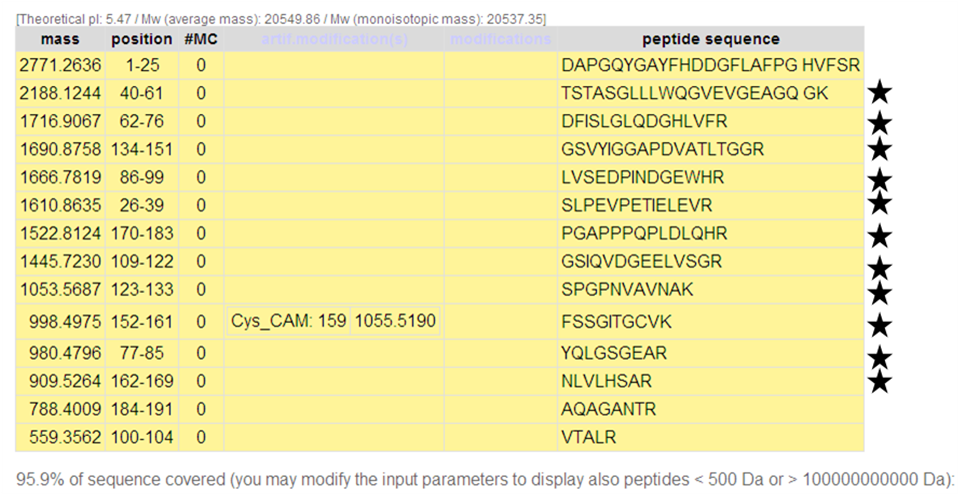


*In silico* trypsin digest was performed on the LG3 peptide using the PeptideMass tool on the ExPASy Proteomics Server. The following options were selected: cysteines treated with iodoacetomide; methionines oxidized with [M+H]^+^; monoisotopic peptides; no allowed missed cleavage; and peptides larger than 500 Da. The MS/MS data (indicated by the stars) fits with the data generated from the computer driven model of enzymatic-digestion. In addition, a BLAST search was performed on the LG3 peptide sequence identified and it was found that no other known protein / peptide shares sequence homology above 88% coverage.
